# Usefulness of Telemedicine for Disabled Children Receiving Feeding Therapy

**DOI:** 10.1007/s00455-022-10482-w

**Published:** 2022-06-29

**Authors:** Fumiyo Tamura, Takeshi Kikutani, Reiko Machida, Tomoko Isoda, Kimiko Hobo, Hiroyuki Yamada, Miho Kodama, Sae Genkai, Miki Mizukami, Yuko Tanaka, Taeko Sakuda, Hiroyasu Furuya, Noriaki Takahashi

**Affiliations:** 1 The Nippon Dental University, Tama Oral Rehabilitation Clinic, Koganei-shi, Tokyo Japan; 2 Division of Rehabilitation for Speech and Swallowing Disorders, The Nippon Dental University Hospital, Tama Oral Rehabilitation Clinic, Koganei-shi, Tokyo Japan

**Keywords:** Telemedicine, Feeding, Disabled children, Dysphagia, Deglutition Disorders, Rehabilitation, COVID-19

## Abstract

We performed a retrospective cohort study using medical records of 374 pediatric patients who visited a university dental clinic specializing in dysphagia rehabilitation in Japan between 2019 and 2020 to clarify the usefulness of telemedicine among disabled children receiving feeding therapy. The primary outcome was the feeding developmental stage confirmed at the final evaluation. Propensity score matching was performed between individuals in two treatment groups (in-person and telemedicine) before the final analysis using patients’ age, sex, primary disease, gross motor function, and feeding developmental stage as covariates. A total of 36 patients were enrolled in each of the in-person and telemedicine groups. The initial evaluation for the propensity score matched population using the χ^2^ test showed no significant difference between the two groups in any parameter. The feeding developmental stage evaluated at the final evaluation using the Wilcoxon signed-rank test significantly improved compared with the stage at the initial evaluation in both groups (in-parson group, *p* = 0.007; telemedicine group, *p* = 0.013). The difference in level achieved at the final evaluation revealed that the most common level was “unchanged,” followed by “improvement by one level” in both groups, indicating that there was no significant difference in the efficacy of feeding therapy between the two groups (*p* = 0.314). Our results show that telemedicine can achieve the same therapeutic outcomes as in-person therapy to improve feeding function in children with disabilities when receiving feeding therapy.

## Introduction

Since 2019, dental practice and education systems have been greatly affected by the COVID-19 pandemic; thus, stringent and workable infection control is needed worldwide [1]. In these circumstances, telemedicine has been reported to be beneficial and has advanced rapidly in reducing the risk of COVID-19 infection. Guidice et al. reported that telemedicine was usable for monitoring all patients, saving costs of treatment and restricting direct human contact, thereby decreasing the risk of COVID-19 infection [2]. Patel at el. proposed that real-time interactive video consultation such as telemedicine would aid in limiting human contact and facilitate the return of routine dental care in the COVID-19 recovery period [3]. Yang et al. reported that online consultation on oral dentistry during the COVID-19 pandemic, has assisted in establishing the distinction between emergency and non-emergency situations, allowing appropriate emergency management measures to be taken [4].

To date, telemedicine has been used for several medical services. Reviews and meta-analyses discovered that telemedicine is effective not only in psychological treatment, such as online delivery of psychological interventions and cognitive behavioral therapy for the treatment of anxiety, but also in physical treatment, such as internet-based physical activity interventions [5]. These studies demonstrate that telemedicine has the potential to play a variety of roles in medical field.

The disadvantages of telemedicine should also be considered. A review of 80 reliable articles on the effectiveness of telemedicine concluded that different kinds of knowledge are needed, including a more economic-focused analysis of telemedicine from the patients’ perspective, and effectiveness and outcome as collaborative achievements [5–8].

Several reports on telemedicine for dysphagia rehabilitation for adult patients have been published [9, 10]. In addition, using telemedicine systems for children with dysphagia has been reported [11, 12]. These findings suggest that telemedicine is still in its initial phase of utilization regarding dysphagia rehabilitation [13]. Therefore, more evidence is required to clarify the benefits of telemedicine compared to in-person therapy and to weigh the potential disadvantages in providing medical care in cases of dysphagia.

Hence, this study aimed to clarify the usefulness of telemedicine in dysphagia rehabilitation, hereafter referred to as “feeding therapy,” for disabled children by comparing their feeding function after in-person versus telemedicine-based therapy.

## Materials and Methods

### Study Design and Participants

A retrospective cohort study of feeding therapy outcomes was performed using the medical records of 374 pediatric patients who visited a university dental clinic specializing in dysphagia rehabilitation in Japan between January 2019 and December 2020.

At baseline, 462 pediatric patients (age ≤ 18 years) who received feeding therapy during the study period were included. The patients presented with their parents or guardians with a chief complaint of dysphagia. Among them, 37 patients who made less than four visits to the clinic for feeding therapy were excluded, and data were collected from the medical records of the remaining 425 patients (231 males and 194 females). The 425 patients were divided into two groups for treatment. The telemedicine group included 37 patients (16 males and 21 females) who completed at least the second and third of four visits through telemedicine. The control group consisted of 388 patients (215 males and 173 females) who received four times of in-person therapy during the same period.

**Fig. 1:**
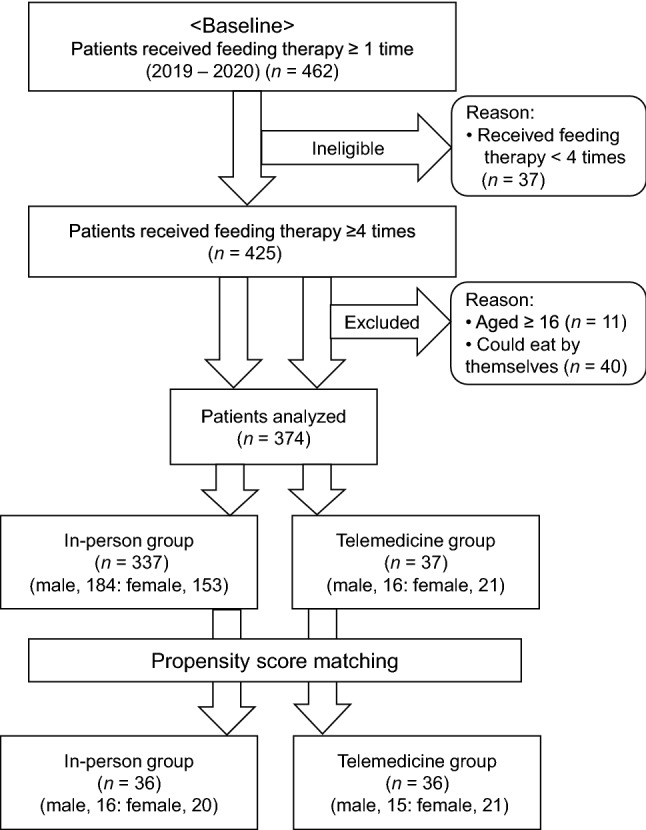
Data selection process for analyses

Among the 425 patients, 51 were excluded (11 patients aged 16 years or older were excluded due to a change in age criteria from age of ≤ 18 years to < 16 years to reduce the number of participants, and 40 patients who could self-feed at the initial developmental stage of feeding function were also excluded). The remaining 374 patients (337 in the in-person group [184 males and 153 females] and 37 in the telemedicine group [16 males and 21 females]) were included in the study (Fig. 1).

### Telemedicine Treatment

Feeding therapy was managed by licensed dentists and dental hygienists specialized in dysphagia rehabilitation.

At the initial visit, the dentist collected information from patients’ parent/guardian about the chief complaint, growth history, past/present medical details, and present illness, and the hygienist enquired about factors related to feeding function. Subsequently, the dentist performed oral examination and palpation of the whole body. For patients capable of oral feeding, dentists and dental hygienists performed external observations of the patient’s eating process.

Based on the results of these examinations, each patient’s developmental stage of feeding function and dysphagia was assessed and diagnosed in accordance with the classifications reported by Ayano et al. [14]. Each patient’s feeding developmental stage and dysphagia symptoms were considered to determine the treatment strategy in terms of feeding posture, food form, feeding assistance method, and feeding training.

For the in-person group, feeding therapy was continued based on the initial treatment strategy. In the telemedicine group, treatment was preceded by at least one in-person therapy session to determine the telemedicine treatment plan. Telemedicine was administered using an online communication application. The communication devices used were a desktop personal computer on the provider’s side and a tablet or mobile phone on the patient’s side. Each telemedicine session started with a question to the parent/guardian about the patient’s feeding function.

After confirming that the patient’s physical condition was stable and giving due consideration to the prevention of aspiration and asphyxiation, the medical provider evaluated the patient’s feeding developmental stage by external observation of the patient during eating and noted dysphagia symptoms. The provider then provided guidance on feeding posture, food form, feeding assistance method, and indirect/direct training method. For patients who did not take food orally, the provider only checked/guided the indirect training method.

### Data Collection

The patient data examined included age, sex, type of main disabilities, gross motor function at the initial evaluation during the study period, and the feeding developmental stage confirmed at the initial and final (fourth) evaluation.

### Type of Main Disabilities

Disabilities were classified into three categories: (1) physical disabilities, including physical disorders such as cerebral palsy, encephalitis, and muscular dystrophy; (2) intellectual disabilities, including non-physical disorders such as chromosomal abnormalities and intellectual dysfunction; and (3) other disabilities with no identifiable organic abnormality or underlying disease.

### Gross Motor Development Milestones

According to the previously reported methods [15–17], we included the following as gross motor development milestones: (1) without steady head control, (2) with steady head control, (3) sitting up, (4) pulling up to standing, (5) aided walking, and (6) unaided walking.

### Developmental Stage of Feeding Function

The patients’ feeding function was evaluated based on established feeding developmental stages [14]: (1) the suckle feeding and pre-feeding period, (2) acquiring the ability to swallow with the lips closed, (3) acquiring the ability to take food with the lips closed, (4) acquiring the ability to push mashed food with the tongue against the anterior hard palate, (5) acquiring the ability to perform mastication, (6) beginning finger feeding, and (7) beginning spoon feeding (Table 1). All participants ate food with the most suitable texture for their developmental stage of feeding function. They used spoons that were most suitable for their developmental stage: thus, the shape of spoons might not have been equal among all the participants.

**Table 1:** Outline of the developmental stage of feeding functions in healthy children

Mean age (in months)	Developmental stage of the feeding functions	Characteristic feeding movement
Before 5 months (Lactation period)	Suckle feeding and pre-feeding period	Primitive reflex (rooting reflex/sucking reflex) Infantile swallowing
5–6 months (Weaning period)	Acquiring the ability to swallow with the lips closed	Slight movement of the mouth
	Acquiring the ability to take food with the lips closed	Low tonus of the orbicularis oris muscle
7–8 months (Weaning period)	Acquiring the ability to push mashed food with the tongue against the anterior hard palate	Simple up-and-down movement of the tongue and mandible
9–11 months (Weaning period)	Acquiring the ability to perform mastication	Complex rotary movements of the tongue and mandible
Over 12 months (After weaning period)	Beginning finger feeding	Can eat food by holding it in the hand and taking it to the mouth
	Beginning spoon feeding	Can eat food with a spoon or fork in the hand

### Outcome Measures

The main outcome was the feeding developmental stage at the final evaluation.

#### Propensity Score Matching

Using data obtained from 374 patients (337 in the in-person group and 37 in the telemedicine group), propensity score matching was performed with age, sex, type of main disabilities, gross motor function, and developmental stage of feeding function as covariates [18].

#### Sample Size Analysis

The sample size was calculated according to a previous study [19] to ensure a power of 70% for type II errors and a significance level at ≤ 10% for type I errors. The effect size was assumed to be medium. G*Power 3.1.9.2 Statistical Power Analyses for Windows was used to estimate the sample size. Based on the calculation, we planned to examine the medical records of more than 92 participants for the analysis.

### Statistical Analysis

For comparisons between the two groups, the t-test and Mann–Whitney U-test were performed for age and the chi-square test and Fisher’s exact test were performed for sex, gross motor function, primary disease, and feeding developmental stage. The Wilcoxon signed-rank test was performed to analyze the change in the feeding developmental stage between the initial and final visits. The chi-square test was performed to compare the effect of feeding therapy on the feeding developmental stage between the groups.

### Ethics

At the initial visit, the parents/guardians of the patients provided oral and written consent to the partial use of the patients’ medical information for the study under the protection of personal information. This study was approved by the Ethics Committee of the Nippon Dental University, School of Life Dentistry (Approval No. NDU-T2020-04).

## Results

After propensity score matching, 36 patients were included in the in-person (16 males and 20 females, mean age: 4.7 ± 3.5 years) and telemedicine groups (15 males and 21 females, mean age: 4.0 ± 2.9 years).

The baseline characteristics of the crude population (*n* = 374) and the propensity score matched population (*n* = 72) are shown in Table 2. The most common developmental stage of feeding function in both groups was the ability to push mashed food with the tongue against the anterior hard palate (17 in the in-person and 14 in the telemedicine groups).

**Table 2:** Patients’ baseline characteristics in the crude population and the propensity score matched population

Characteristics	Crude population			*P* value	Propensity score matched population			*P* value
	Overall (*n* = 374)	In-person (*n* = 337)	Telemedicine (*n* = 37)		Overall (*n* = 72)	In-person (*n* = 36)	Telemedicine (*n* = 36)	
Mean age, years (± standard deviation)	4.6 (3.4)	4.6 (3.5)	4.0 (2.9)	N.S	4.4 (3.2)	4.7 (3.5)	4.0 (2.9)	N.S
Sex, *n* (%)								
Male	200 (53.5)	184 (54.6)	16 (43.2)	N.S	31 (43.1)	16 (44.4)	15 (41.7)	N.S
Female	174 (46.5)	153 (45.4)	21 (56.8)		41 (56.9)	20 (55.6)	21 (58.3)	
Gross motor development milestones, *n* (%)								
Without steady head control	33 (8.8)	26 (7.7)	7 (18.9)	N.S	10 (13.9)	4 (11.1)	6 (16.7)	N.S
With steady head control	65 (17.4)	63 (18.7)	2 (5.4)		4 (5.6)	2 (5.6)	2 (5.6)	
Sitting up	65 (17.4)	58 (17.2)	7 (18.9)		13 (18.1)	6 (16.7)	7 (19.4)	
Pulling up to standing	28 (7.5)	27 (8.0)	1 (2.7)		1 (1.4)	0 (0)	1 (2.8)	
Aided walking	29 (7.8)	27 (8.0)	2 (5.4)		3 (4.2)	1 (2.8)	2 (5.6)	
Unaided walking	154 (41.2)	136 (40.4)	18 (48.6)		41 (56.9)	23 (63.9)	18 (50.0)	
Type of main disabilities								
Physical disability	105 (28.1)	93 (27.6)	12 (32.4)	0.02^*^	18 (25.0)	7 (19.4)	11 (30.6)	N.S
Intellectual disability	251 (67.1)	231 (68.5)	20 (54.1)		42 (58.3)	22 (61.1)	20 (55.6)	
Others	18 (4.8)	13 (3.9)	5 (13.5)		12 (16.7)	7 (19.4)	5 (13.9)	
Development of feeding functions, *n* (%)								
Suckle feeding and pre-feeding period	70 (18.7)	65 (19.3)	5 (13.5)	N.S	8 (11.1)	3 (8.3)	5 (13.9)	N.S
Acquiring the ability to swallow with lip closed	34 (9.1)	32 (9.5)	2 (5.4)		3 (4.2)	1 (2.8)	2 (5.6)	
Acquiring the ability to take food with lips closed	47 (12.6)	40 (11.9)	7 (18.9)		12 (16.7)	6 (16.7)	6 (16.7)	
Acquiring the ability to push the mashed food with tongue against anterior hard palate	149 (39.8)	135 (40.1)	14 (37.8)		31 (43.1)	17 (47.2)	14 (38.9)	
Acquiring the ability to perform mastication	74 (19.8)	65 (19.3)	9 (24.3)		18 (25.0)	9 (25.0)	9 (25.0)	

*NS* not significant

*p < 0.05 was considered statistically significant

In the crude population, significant differences were observed between the in-person and telemedicine groups in the type of main disability (*p* = 0.02), but not in other parameters, including age, sex, gross motor function, and feeding developmental stage. In the propensity score matched population, the initial evaluation using the χ^2^ test showed no significant difference between the two groups in any parameter.

Tables 3 and 4 show changes in the developmental stage of feeding function between the initial and final evaluations in the propensity score matched population according to the treatment group. The most common improvement in the developmental stage was observed in 12 (33.3%) and 9 (25.0%) patients in the in-person and telemedicine groups, respectively. Of them, 9 (25.0%) in the in-person group and 6 (16.7%) in the telemedicine group acquired the ability to push the mashed food with the tongue against the anterior hard palate.

**Table 3:** Change in the developmental stage of feeding function between the initial and final evaluation in the in-person group of the propensity score matched population

		Developmental stage at final evaluation						
		Suckle feeding and pre-feeding period	Acquiring the ability to swallow with the lip closed	Acquiring the ability to take food with the lips closed	Acquiring the ability to push mashed food with the tongue against the anterior hard palate	Acquiring the ability to perform mastication	Beginning spoon feeding	Total (*n*)
Developmental stage at initial evaluation	Suckle feeding and pre-feeding period	2	0	1	0	0	0	3
	Acquiring the ability to swallow with the lips closed	0	1	0	0	0	0	1
	Acquiring the ability to take food with the lips closed	0	1	3	1	1	0	6
	Acquiring the ability to push mashed food with the tongue against the anterior hard palate	0	0	0	8	8	1	17
	Acquiring the ability to perform mastication	0	0	0	1	8	0	9
Total (*n*)		2	2	4	10	17	1	36

Statistical analysis showed significant improvement at the final evaluation compared with the initial evaluation (*p* = 0.007; Wilcoxon signed-rank test)

**Table 4:** Changes in the developmental stage of feeding function between the initial and final evaluation in the telemedicine group of the propensity score matched population

		Developmental stage at final evaluation							
		Suckle feeding and pre-feeding period	Acquiring the ability to swallow with the lip closed	Acquiring the ability to take food with the lips closed	Acquiring the ability to push mashed food with the tongue against the anterior hard palate	Acquiring the ability to perform mastication	Beginning finger feeding	Beginning spoon feeding	Total (*n*)
Developmental stage at initial evaluation	Suckle feeding and pre-feeding period								
	Acquiring the ability to swallow with the lip closed	1	1	0	0	0	0	0	2
	Acquiring the ability to take food with the lips closed	0	0	4	2	0	0	0	6
	Acquiring the ability to push mashed food with the tongue against the anterior hard palate	0	0	0	8	5	1	0	14
	Acquiring the ability to perform mastication	0	0	0	0	8	0	1	9
Total (*n*)		6	1	4	10	13	1	1	36

Statistical analysis showed significant improvement at the final evaluation compared with the initial evaluation (*p* = 0.013; Wilcoxon signed-rank test)

The second most common developmental stage was the ability to take food with the lips closed. In each treatment group, two of six patients in this stage acquired a higher level of feeding function at the final evaluation. Statistical analysis showed significant improvement in the feeding developmental stage at the final evaluation compared with at the initial evaluation in both groups (in-person group, *p* = 0.007; telemedicine group, *p* = 0.013; Wilcoxon signed-rank test).

The change in the developmental stage of feeding function from the initial to final evaluation was compared between the in-person and telemedicine groups. The difference in feeding function was indicated by “ − 1” per level of worsened function, “0” for unchanged function, and “ + 1” per level of improved function (Table 5). The most common difference in the feeding developmental stage at the final evaluation was “0” (unchanged function) in both groups (22 of 36 [61.1%] patients in the in-person group and 26 of 36 [72.2%] in the telemedicine group).

**Table 5:** Changes in the developmental stage of feeding function before and after feeding therapy in the propensity score matched population

	Difference level	In-person group	Telemedicine group	Total (*n*)
Difference in the developmental stage	− 1	2	1	3
	0	22	26	48
	1	9	7	16
	2	2	0	2
	3	0	2	2
	4	1	0	1
Total (*n*)		36	36	72

The chi-square test showed no significant difference between the two groups (*p* = 0.314)

The second most common difference was “1,” indicating improvement by one level from the initial to final evaluation, reported by 9 (25.0%) patients in the in-person group and 7 (19.4%) in the telemedicine group. The chi-square test showed no significant difference between the two groups (*p* = 0.314).

## Discussion

To the best of our knowledge, this is the first study to demonstrate the usefulness of telemedicine for patients with dysphagia. The current study provides useful information for disabled children with dysphagia since telemedicine-based feeding therapy is expected to be more convenient for patients with an impaired ability to perform activities of daily living.

Several studies have reported the advantages of using telemedicine for dysphagia rehabilitation. Wall et al. investigated the effectiveness of dysphagia rehabilitation in patients with head and neck cancer undergoing curative chemoradiotherapy using the application “Swallow IT” [9]. According to Malandraki et al., using asynchronous teleconsultation performed by such as a trained clinician who completed a videofluoroscopic swallowing study in Greece and an expert speech and language pathologist in the USA can result in a higher quality of care for patients with dysphagia [20]. Furthermore, several telemedicine studies have been conducted in children with dysphagia. Clawson et al. investigated the need for video teleconferencing for children with dysphagia, family satisfaction, provider satisfaction, and clinical outcomes [11]. Raatz et al. indicated the effect of modified videoconferencing for conducting feeding assessments in pediatric patients with dysphagia in the home via telepractice [12].

While these factors suggest the benefits of telemedicine in dysphagia rehabilitation, its disadvantages should also be considered. Hjelm et al. stated that the anticipated disadvantages of telemedicine include the breakdown between healthcare professionals and patients as well as among healthcare professionals, matters related to the quality of health information, and organizational and bureaucratic difficulties [6]. Spear et al. reported that the disadvantages of telemedicine for patients with Parkinson’s disease include lack of hands-on care, lack of intimacy, and technical difficulties [7]. Funderskov et al. demonstrated some advantages of using telemedicine in palliative care and pointed out that video consultation has potential barriers related to patients’ privacy [8].

Children who require feeding therapy include those with physical disabilities such as cerebral palsy and muscular dystrophy, those with intellectual disabilities such as chromosomal abnormalities, those with severe motor and intellectual disabilities [21], and those with autism or other mild developmental disabilities. Severely disabled children are prone to obstructive and restrictive respiratory disorders, have difficulty clearing sputum from the airways, and are prone to multiple organ disorders such as muscle weakness, muscle spasticity, seizure, and gastroesophageal reflux, possibly leading to respiratory failure [22, 23].

Children receiving severe medical care often require feeding therapy. In recent studies from Japan, an increasing number of children are reportedly in need of severe medical care, such as mechanical ventilation and tube feeding [24]. This includes not only children with severe motor and intellectual disabilities, but also those who are able to walk (move) while receiving medical care, although many of them have concomitant respiratory diseases and are susceptible to infection [21–23].

Due to their susceptible state, many parents/guardians report that they want to avoid taking their children outside as much as possible. Among patients with mild physical disabilities without respiratory diseases, such as those with intellectual disabilities or those with autism, there also are cases in which in-person therapy is difficult on an outpatient basis. For example, these children often become nervous or distracted during outpatient visits at medical institutions since the setting is not part of their daily lives, making it difficult to administer effective feeding therapy. Telemedicine may be an effective means of solving such problems.

The standard feeding therapy program at our clinic involves external observations, screening tests, and detailed examinations. Based on these results, the patient’s developmental stage of feeding function is determined and subsequent treatments are provided, including guidance on food environment and nutrition, as well as feeding training (indirect and direct). International guidebooks are available for feeding therapy in children [25, 26]. In Japan, the methods for feeding therapy for disabled children described by Kaneko et al. have been adopted as the standard guidelines [27].

In feeding therapy for children, however, observation and evaluation of eating scenes are of particular importance [26]. Telemedicine allows the observation of a patient’s eating ability in a daily setting. In outpatient treatment, it is often the case that patients cannot demonstrate their full abilities because the situation is different from that of their daily lives, and they are required to eat under supervision. Telemedicine allows children and their family members to eat in a relaxed state in a familiar environment. While performing telemedicine, it is important to remember that is not applicable to detailed examinations, such as videofluoroscopic examinations and videoendoscopic evaluation of swallowing or functional training that requires guidance through body contact.

After completing the feeding therapy program in this study, there was no significant difference between the in-person and telemedicine groups regarding change in the feeding developmental stage from the initial to final evaluation, suggesting that children in both groups acquired feeding function at the same pace. There was also no difference between the two groups regarding the change in developmental stage between the initial and final evaluation. In both groups, the most common developmental stage was the ability to push mashed food with the tongue against the anterior hard palate, which in many patients improved to the ability to perform mastication after completing feeding therapy. This suggests that the therapy strategy aimed at acquiring the ability to perform mastication was most effective in both in-person and telemedicine settings.

In feeding training for children, methods to improve mastication change with the patient’s developmental stage and include exercising the oral muscles (through massaging), training in basic chewing skills, and changing the diet to include slightly firmer food for chewing with the molar teeth [28]. These training methods are communicated by dentists or dental hygienists to parents/guardians verbally or using a manual with illustrations. The treatment is then administered by the parents/guardians, requiring no physical contact by the healthcare providers. Therefore, our results indicate that telemedicine was also effective in promoting improved feeding function, as long as the patients are capable of oral feeding.

The results of this study showed that, despite various individual and baseline characteristics, many patients in both in-person and telemedicine groups had improved feeding developmental stage. In telemedicine-based feeding therapy, a preceding in-person examination, diagnosis, and treatment planning, as well as direct guidance, are crucial. Based on our current study results, future guidelines should be developed to provide appropriate feeding therapy through telemedicine.

A limitation of this study is that it was conducted at only two affiliated medical institutions. While this had the advantage of maintaining the quality of the content of feeding therapy, we plan to conduct a multicenter study to clarify the efficacy of telemedicine-based feeding therapy in the future. Another limitation is that none of the patients who were incapable of oral feeding showed improvement in the feeding developmental stage through telemedicine, suggesting that telemedicine may not be effective in patients with severe dysphagia. This issue should be addressed in further studies with a larger sample size. Finally, the rapid increase in the use of telemedicine is largely due to the need to mitigate the spread of COVID-19. Although the COVID-19 pandemic will subside in the near future [29], it cannot be ruled out that similar infectious disease outbreaks can occur in the future. To prepare for such events, it is important to explore methods to provide high-quality telemedicine.

## Conclusion

Our results show that telemedicine-based feeding therapy can achieve the same therapeutic outcomes as in-person therapy. Telemedicine is expected to be increasingly used in dysphagia rehabilitation.
